# Supplementary material for: Pupil-linked phasic arousal evoked by violation but not emergence of regularity within rapid sound sequences
Source: Nat Commun. 2019 Sep 6;10:4030. doi: 10.1038/s41467-019-12048-1 (PMC6731273; doi:10.1038/s41467-019-12048-1)
Supplement: Supplementary file 1 — Supplementary Information [file 41467_2019_12048_MOESM1_ESM.pdf]

Supplementary Materials

Pupil-linked phasic arousal evoked by violation but not emergence of regularity within rapid sound sequences

Sijia Zhao[1], Maria Chait[1], Fred Dick[2,3], Peter Dayan[4], Shigeto Furukawa[5], Hsin-I Liao[5]

[1] Ear Institute, University College London, London WC1X 8EE, UK
[2] Department of Psychological Sciences, Birkbeck College, London, WC1E 7HX
[3] Department of Experimental Psychology, University College London, WC1H 0DS
[4] Max Planck Institute for Biological Cybernetics, 72076 Tübingen, Germany
[5] NTT Communication Science Laboratories, NTT Corporation, Atsugi, 243-0198 Japan

Corresponding Author:
Maria Chait
m.chait@ucl.ac.uk
Ear Institute, University College London
332 Gray's Inn Road, London WC1X 8EE, UK

Supplementary Audio 1-4: We provide examples of the 4 main experimental conditions in Experiment 1 (REG10-RAND20; RAND20; RAND20-REG10; REG10). See Figure 1 in the main text for more information. Further stimulus examples for each of the reported experiments are available at http://dx.doi.org/10.5522/04/c.4590887.

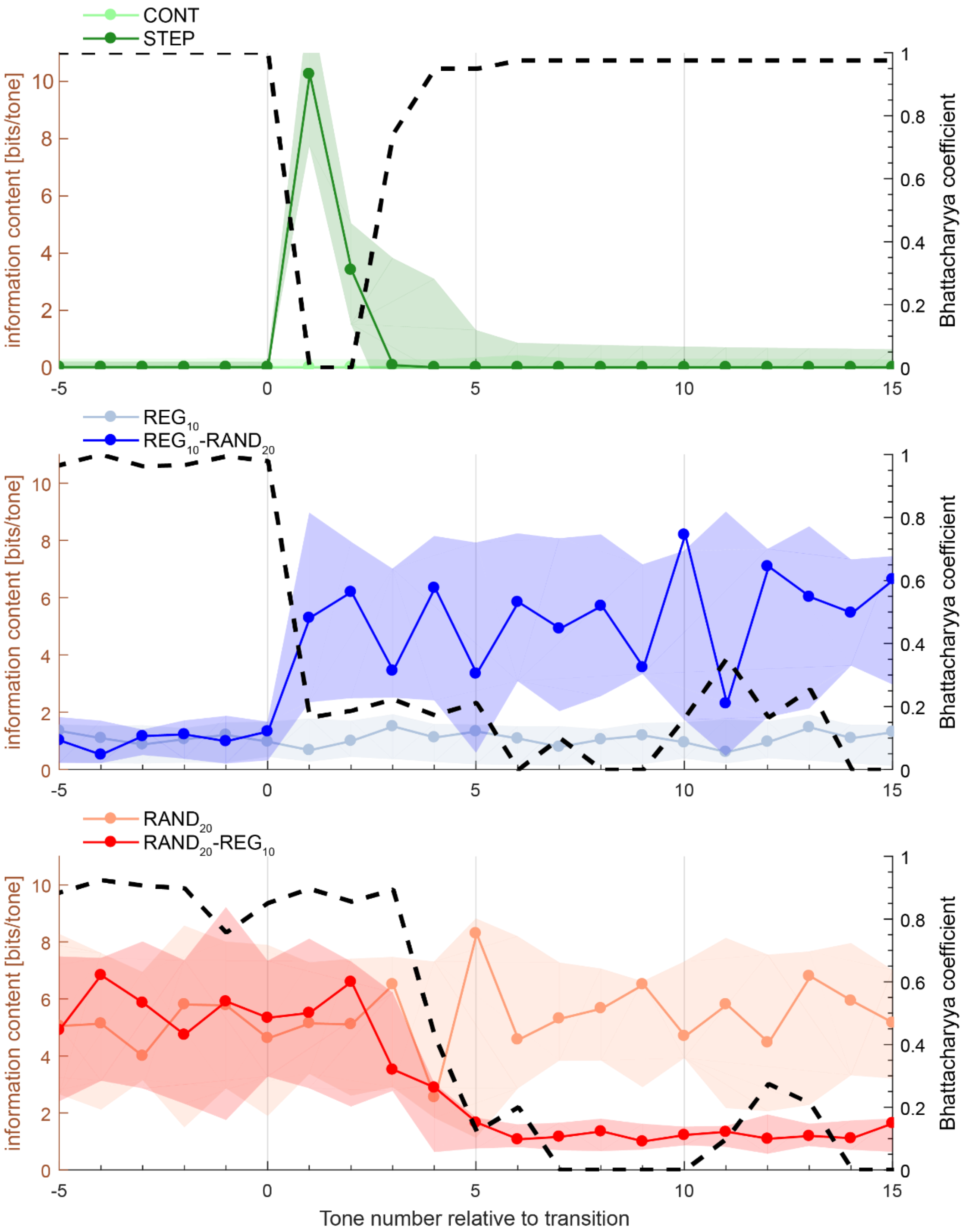

CONT
STEP
REG10
REG10-RAND20
RAND20
RAND20-REG10
information content [bits/tone]
Bhattacharyya coefficient
Tone number relative to transition

Supplementary Figure 1: Modelling results for the stimulus set in Experiment 1A. To quantify the predictability of each tone-pip within the sequences, we applied a model of auditory expectancy (Information Dynamics Of Music; IDyOM[1]) as a theoretical benchmark. The model is based on multiple-viewpoint variable-order Markov chains; for each tone in a sequence, it outputs "information content" (IC) as a measure of unexpectedness, given the preceding context. This model is sensitive to sequential regularities and is hence a suitable model for quantifying the statistics of the present stimuli. It was also previously used in the context of an MEG study based on the same stimuli[2]. We applied IDyOM to the stimuli in Experiment 1A; it was ran on the entire experimental session, with stimuli in different conditions presented in a random order (in the same way they were delivered to the human participants). We used the LTM+ model configuration, which initiates with an empty model, then learns over the stimulus set, updating the model after each tone. This mirrors the experience of the human participants who likely learned the probability structure of the stimuli throughout the course of the experimental session. Plotted is the information content associated with an example single trial (always the last trial for each of the stimulus categories; therefore, the output reflects the probability associated with each tone in light of the most complete internal model). To focus on the transition period, we plot the interval from 5 tones before the transition to 15 tones after the transition. As in Figure 1 in the main text, in $RAND_{20}$-REG_{10} sequences, the transition time is defined as occurring after the first full regular cycle, i.e. once the transition becomes theoretically detectable. Shading indicates 2 standard deviations from the average across all trials. The black dashed curve indicates the Bhattacharyya coefficient—an estimate of the amount of overlap between the two distributions—labelled on the right y-axis. BC=1 indicates perfect overlap BC=0 indicates zero overlap. For STEP and REG_{10}-$RAND_{20}$ the model shows an effectively instantaneous detection of the transition. For in $RAND_{20}$-REG_{10}, the model requires about 4-5 tones (after the effective transition) to discover the emergence of regularity. Intriguingly, active listeners (Experiment 3A) show comparable performance - requiring 277ms (5.5 tones) to detect the transition in $RAND_{20}$-REG_{10}.

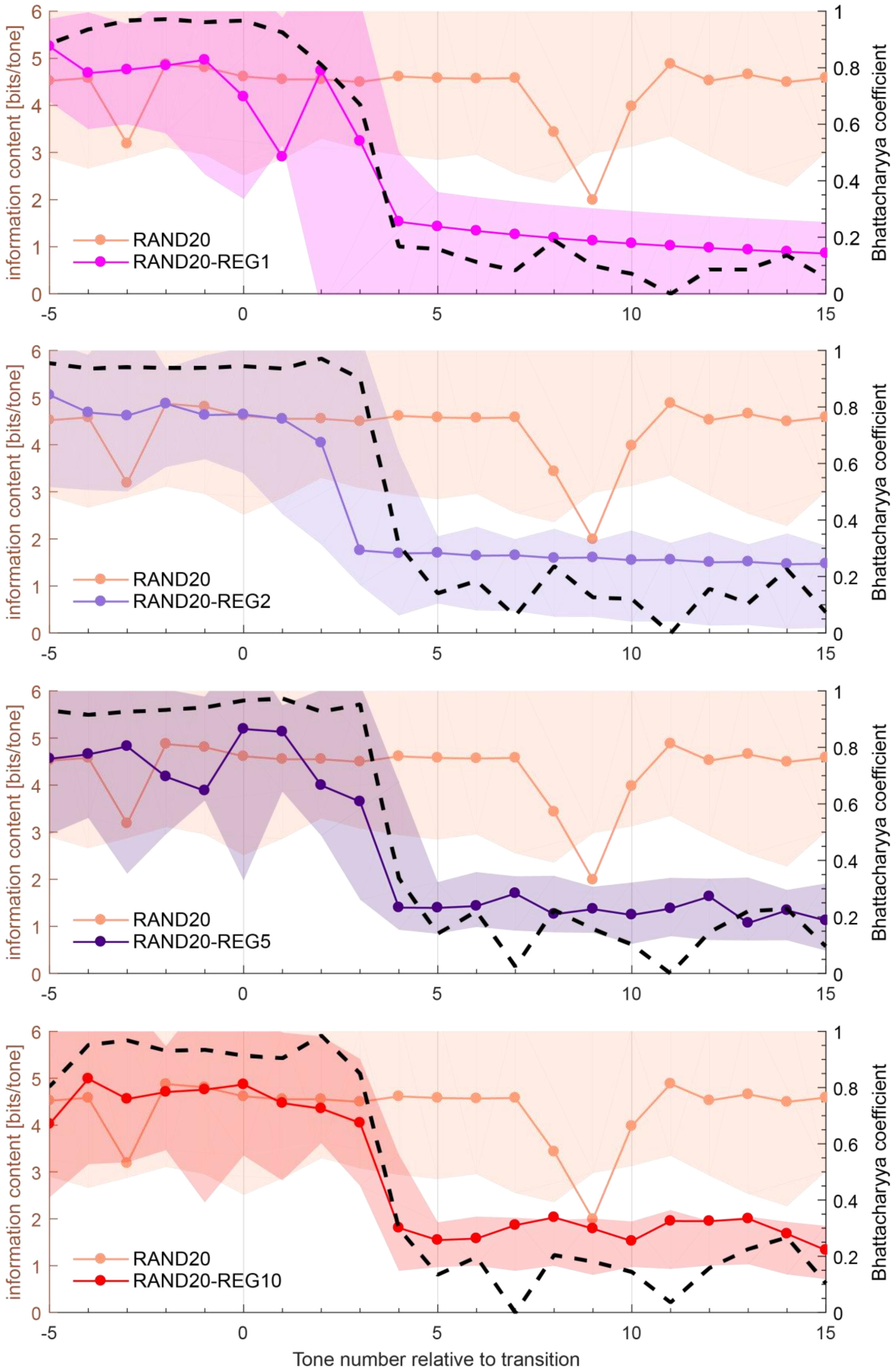
information content [bits/tone]
Bhattacharyya coefficient
RAND20
RAND20-REG1
RAND20-REG2
RAND20-REG5
RAND20-REG10
Tone number relative to transition

Supplementary Figure 2: Modelling results (as described for Supplementary Figure 1, above) for the stimulus set in Experiment 4B: $RAND_{20}$-REG_{1}, $RAND_{20}$-REG_{2}, $RAND_{20}$-REG_{5}, $RAND_{20}$-REG_{10} and their control $RAND_{20}$. The information content for each tone from the chosen single trial (always the last presentation of each condition within the experimental session) is shown against the tone number relative to transition. To focus on the transition period, we plot the interval from 5 tones before the transition to 15 tones after the transition. As in Figure 1, in the main text, the transition time is defined as occurring after the first full regularity cycle. Shading indicates 2 standard deviations from the average across all trials. The black dashed curve indicates the Bhattacharyya coefficient—an estimate of the amount of overlap between the two distributions—labelled on the right y-axis. All transition conditions show a gradual decline in IC over several tones, though the drop in $RAND_{20}$-REG_{1} appears to occur one tone earlier, on average. The temporary dip in IC around tone#9 in the $RAND_{20}$ condition reflects an idiosyncrasy of the single trial plotted (note that the same trial is replotted in all sub-panels because it was the last $RAND_{20}$ trial presented) and reflects the fact that the sequence happened to contain an arrangement of tones that occurred previously in another trial and was therefore familiar to the model.

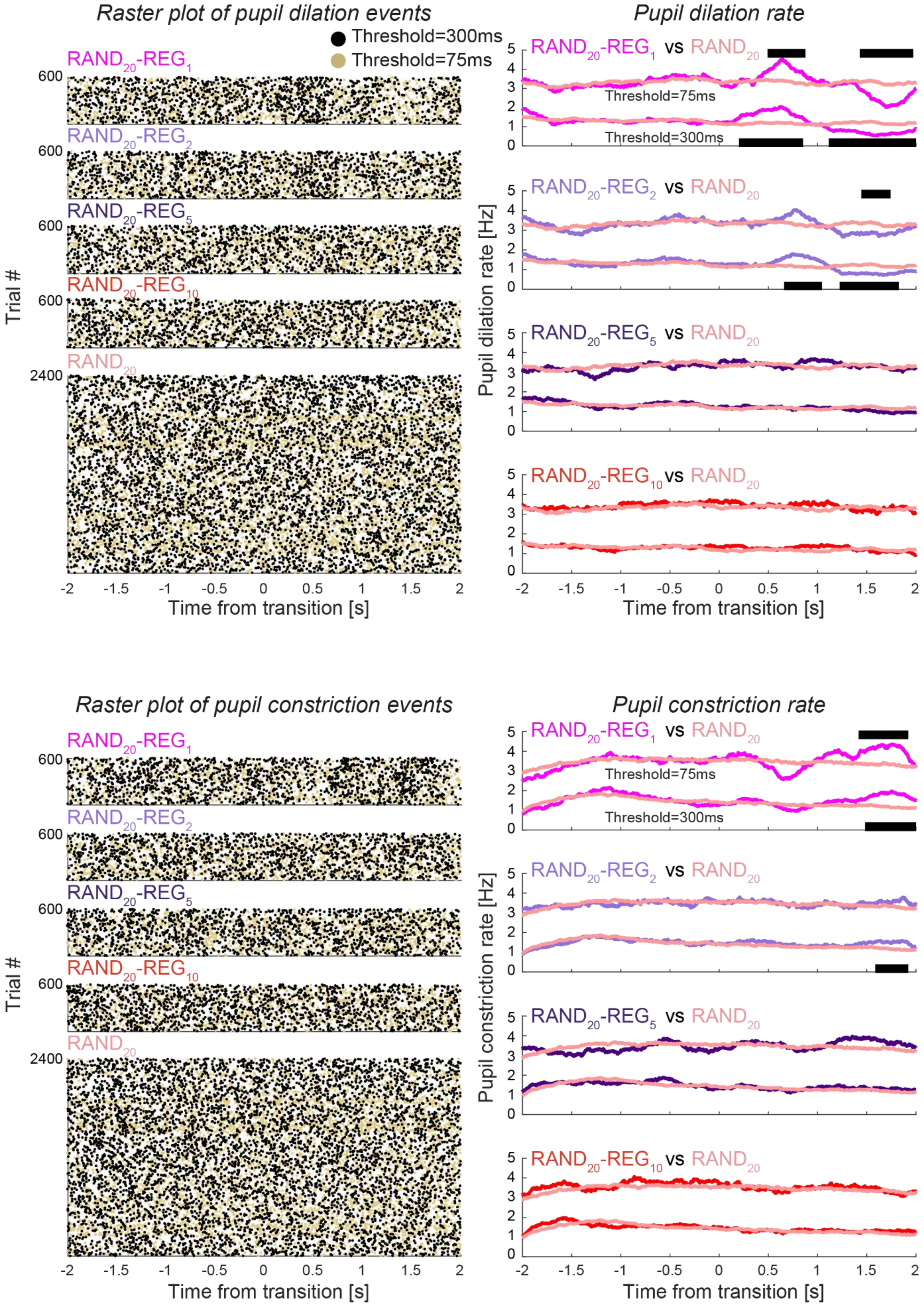
Raster plot of pupil dilation events
Threshold=300ms
Threshold=75ms
RAND20-REG1
RAND20-REG2
RAND20-REG5
RAND20-REG10
RAND20
Trial #
Time from transition [s]
Pupil dilation rate
RAND20-REG1 vs RAND20
RAND20-REG2 vs RAND20
RAND20-REG5 vs RAND20
RAND20-REG10 vs RAND20
Threshold=75ms
Threshold=300ms
Pupil dilation rate [Hz]
Time from transition [s]
Raster plot of pupil constriction events
Pupil constriction rate
Pupil constriction rate [Hz]

Supplementary Figure 3: Experiment 4B: Pupil dilation and constriction rates. [Top left] Raster plots of pupil dilation (PD) events extracted from all trials and all participants (collapsed over the two groups, N = 30). Each line represents a single trial. Black dots represent the onset of a pupil dilation with a duration of at least 300ms, yellow dots represent pupil dilation onsets with a threshold duration of 75ms. Transition time is indicated by a black vertical line. [Top right] Pupil dilation rate (running average with a 500ms window) as a function of time relative to the transition. From top to bottom, one of four transition conditions are plotted against the no-change control $RAND_{20}$: $RAND_{20}$-REG_1, $RAND_{20}$-REG_2, $RAND_{20}$-REG_5 and $RAND_{20}$-REG_{10}. The black horizontal lines indicate time intervals where cluster-level statistics showed significant differences between each change condition and the no-change control $RAND_{20}$. The statistics for the PD rates with a threshold duration of 75ms and 300ms are placed above and below the graph, respectively. The lower panels present the pupil constriction (PC) rate results, arranged in the same format.

References

1. Pearce, M. T. The construction and evaluation of statistical models of melodic structure in music perception and composition. (City University London, 2005).
2. Barascud, N., Pearce, M. T., Griffiths, T. D., Friston, K. J. & Chait, M. Brain responses in humans reveal ideal observer-like sensitivity to complex acoustic patterns. *Proc. Natl. Acad. Sci.* 201508523 (2016). doi:10.1073/pnas.1508523113
