# Supplementary material for: Pupil-linked phasic arousal evoked by violation but not emergence of regularity within rapid sound sequences
Source: Nat Commun. 2019 Sep 6;10:4030. doi: 10.1038/s41467-019-12048-1 (PMC6731273; doi:10.1038/s41467-019-12048-1)
Supplement: Supplementary file 3 — Reporting Summary [file 41467_2019_12048_MOESM3_ESM.pdf]

nature research

Corresponding author(s): Maria Chait

Last updated by author(s): Jul 29, 2019

Reporting Summary

Nature Research wishes to improve the reproducibility of the work that we publish. This form provides structure for consistency and transparency in reporting. For further information on Nature Research policies, see Authors & Referees and the Editorial Policy Checklist .

Statistics

For all statistical analyses, confirm that the following items are present in the figure legend, table legend, main text, or Methods section.

n/a	Confirmed	
☐	☒	The exact sample size (*n*) for each experimental group/condition, given as a discrete number and unit of measurement
☐	☒	A statement on whether measurements were taken from distinct samples or whether the same sample was measured repeatedly
☐	☒	The statistical test(s) used AND whether they are one- or two-sided<br>*Only common tests should be described solely by name; describe more complex techniques in the Methods section.*
☐	☒	A description of all covariates tested
☐	☒	A description of any assumptions or corrections, such as tests of normality and adjustment for multiple comparisons
☐	☒	A full description of the statistical parameters including central tendency (e.g. means) or other basic estimates (e.g. regression coefficient) AND variation (e.g. standard deviation) or associated estimates of uncertainty (e.g. confidence intervals)
☐	☒	For null hypothesis testing, the test statistic (e.g. *F*, *t*, *r*) with confidence intervals, effect sizes, degrees of freedom and *P* value noted<br>*Give P values as exact values whenever suitable.*
☒	☐	For Bayesian analysis, information on the choice of priors and Markov chain Monte Carlo settings
☒	☐	For hierarchical and complex designs, identification of the appropriate level for tests and full reporting of outcomes
☐	☒	Estimates of effect sizes (e.g. Cohen's *d*, Pearson's *r*), indicating how they were calculated

Our web collection on statistics for biologists contains articles on many of the points above.

Software and code

Policy information about availability of computer code

Data collection	As detailed in the methods section: Stimulus presentation and response recording were controlled with the Psychtoolbox package (Psychophysics Toolbox Version 363) in MATLAB (R2017a; The MathWorks, Inc.).
Data analysis	As detailed in the methods section: MATLAB (R2017a; The MathWorks, Inc.)

For manuscripts utilizing custom algorithms or software that are central to the research but not yet described in published literature, software must be made available to editors/reviewers. We strongly encourage code deposition in a community repository (e.g. GitHub). See the Nature Research guidelines for submitting code & software for further information.

Data

Policy information about availability of data

All manuscripts must include a data availability statement. This statement should provide the following information, where applicable:

- Accession codes, unique identifiers, or web links for publicly available datasets
- A list of figures that have associated raw data
- A description of any restrictions on data availability

Stimulus examples as well as all raw and processed data and the analysis code to reproduce the figures, are available at [http://dx.doi.org/10.5522/04/c.4590887]. A reporting summary for this Article is available as a Supplementary Information file.

Field-specific reporting

Please select the one below that is the best fit for your research. If you are not sure, read the appropriate sections before making your selection.

Life sciences study design

All studies must disclose on these points even when the disclosure is negative.

Sample size	Experiment 1A: Data from 18 participants (11 females; aged 20–29, average 23.41) are presented. Data from one additional participant were excluded due to failure to complete the experiment. Two further participants were excluded due to high blink rates in the STEP condition.<br>Experiment 1B: Data from 14 new participants (13 females; aged 22-26, average 23.1) were used in the analysis. Five additional participants were excluded due to high blinks rates. One further participant was excluded due to poor behavioral performance (0% gap detection hit rate in REG sequences).<br>Experiment 2: Data from 18 new participants (15 females; aged 20-35, average 25.1) are reported. Two additional participants were excluded: one due to high blink rates, and one due to wandering gaze.<br>Experiment 3A: Data from 14 participants (10 females; aged 22–30, average 24.3) are presented. None were excluded.<br>Experiment 3B: Data from 14 participants (12 females; aged 20-31, average 23.9) are presented. None were excluded.<br>Experiment 4A: Data from 12 new participants (9 females; aged 21 - 26, average 23.6) are presented. None were excluded.<br>Experiment 4B: This experiment was performed twice; a total of 30 new participants took part, with 15 participants initially (11 females; aged 20–29, average 23.5) and a new group of 15 participants subsequently (14 females; aged 20–25, average 22.5) to replicate the results of the first cohort. None were excluded.<br>As a confirmation of the adequacy of sample-sizes, we quantified effect sizes in Expt1A and Exp1B, which were chronologically the first and second experiments in this project, and used those values for a power analysis to confirm that N in the subsequent experiments (2-4) was adequate. The power analysis was conducted in the G*Power software package 59, with the following settings: $1 - \beta > 0.8$, and $p = 0.05$.<br>To produce a measure of the magnitude of the PDR effect in Exp1A, B, we first found the latency of the PDR peak in the grand average for STEP and REG-RAND respectively (Figure 2A, B), and used this value to obtain, for each subject, the amplitude in the transition conditions and their respective controls. A net effect measure (quantifying the size of the PDR) was then computed for each subject by taking the difference between each transition and its control (no-change) condition. Note that this manner of reducing the PDR effect to a single number per subject is necessarily more conservative than the time-sensitive analysis employed to quantify the PDR in the main analysis. Both Exp1A and Exp1B enjoyed large effects sizes: Cohen's d~1.2-1.7 for STEP and d=0.7-0.8 for RAND-REG. An overall estimated effect size of 0.8 was therefore fed into the power analysis and yielded an N=12, confirming that all further experiments (Experiment 2-4; N>=12 for all) were adequately powered.
Data exclusions	See exclusion criteria in the text: The following exclusionary criteria were consistently applied across all experiments: To ensure that observed changes in pupil diameter were not blink-related artifacts, participants were excluded if they blinked in more than 50% of trials. Additionally, participants were excluded if their mean gaze location exceeded three standard deviations from the group mean.
Replication	All of our experiments contain an element of self replication, such that the major effects are replicated across multiple experiments.
Randomization	Random allocation.
Blinding	Blinding not relevant to present study because all designs are repeated measures.

Reporting for specific materials, systems and methods

We require information from authors about some types of materials, experimental systems and methods used in many studies. Here, indicate whether each material, system or method listed is relevant to your study. If you are not sure if a list item applies to your research, read the appropriate section before selecting a response.

Materials & experimental systems

n/a	Involved in the study	
☒	☐	Antibodies
☒	☐	Eukaryotic cell lines
☒	☐	Palaeontology
☒	☐	Animals and other organisms
☐	☒	Human research participants
☒	☐	Clinical data

Methods

n/a	Involved in the study	
☒	☐	ChIP-seq
☒	☐	Flow cytometry
☒	☐	MRI-based neuroimaging

Human research participants

Policy information about studies involving human research participants

Population characteristics	See detailed under " sample size" above.
Recruitment	Participants were recruited through UCL's standard subject pool.
Ethics oversight	The experimental procedures were approved by the Research Ethics Committee of University College London. Participants were provided written informed consent and were paid for their participation.

Note that full information on the approval of the study protocol must also be provided in the manuscript.
