# Supplementary material for: Pupil-linked phasic arousal evoked by violation but not emergence of regularity within rapid sound sequences
Source: Nat Commun. 2019 Sep 6;10:4030. doi: 10.1038/s41467-019-12048-1 (PMC6731273; doi:10.1038/s41467-019-12048-1)
Supplement: Supplementary file 4 — Description of Additional Supplementary Files [file 41467_2019_12048_MOESM4_ESM.pdf]

Description of Additional Supplementary Files

File Name: Supplementary Audio 1
Description: Example of a RAND20 stimulus (wav file)

File Name: Supplementary Audio 2
Description: Example of a RAND20-REG10 stimulus (wav file)

File Name: Supplementary Audio 3
Description: Example of a REG10 stimulus (wav file)

File Name: Supplementary Audio 4
Description: Example of a REG10-RAND20 stimulus (wav file)
